# Exploring safety capacity from a risk and safety information integration perspective: Connotation, dimension mining and dimensionality reduction

**DOI:** 10.1016/j.heliyon.2023.e21728

**Published:** 2023-11-04

**Authors:** Fang Yan, Xuan Li, Bing Wang, Youxian Xie, Chao Wu

**Affiliations:** a School of Resources and Safety Engineering, Central South University, Changsha, 410083, PR China; b Safety & Security Theory Innovation and Promotion Center (STIPC), Central South University, Changsha, 410083, PR China

**Keywords:** Safety capacity, Safety similarity theory, Dimension mining, Dimensionality reduction, System safety, Risk analysis

## Abstract

With the development of safety science, the concept of safety capacity (SC) has been proposed. SC has been applied in many fields. Concurrently, there has been an active and robust development of research pertaining to it. However, SC research mainly focuses on practical engineering problems, and SC research is only regarded as the establishment of a quantitative model for system safety. Hence, the theoretical study of SC is ignored. Indeed, the integrated theoretical system and connotation of SC are lacking. In this study, an attempt is made to enrich the theoretical connotation of SC. The following aspects of theoretical exploration are included: (a) The theoretical connotation of SC is redefined so that the lack of theory with respect to SC can be made up. Moreover, research directions of SC are indicated as dimension mining (DM) and dimensionality reduction (DR). (b) The application value of safety similarity theory in the DM of SC is expounded according to the principle of analogism. Core principles of DM are proposed and explained. (c) Common methods of DR are summarized, and the one-sided cognition of DR for the current SC research is corrected. A case study of an explosion accident caused by a boiler water shortage is employed for further discussion. For the investigation of the explosion accident using fault tree analysis (FTA), DR of SC is utilized to provide a more comprehensive explanation. Therefore, the proposed SC is proven to be practical for the analysis of system safety. The results obtained from this study have important implications for research and practice of SC.

## 1 Introduction

### 1.1 Background

Recently, safety science has progressively become a new field of cross-disciplines along with the development of safety science theory [1]. The importance of safety science has received widespread attention and recognition [2]. For instance, the safety science and engineering major was listed as the primary discipline in the Inventory of Disciplinary Categories for the Degree Granting and Talent Cultivation in China on October 1, 2011 [3]. It is important to supplement and improve the corresponding theories of safety science [4], including risk engineering and safety systems. For instance, the research and development of safety information-based accident causation theory is beneficial for the analysis and prevention of major accidents [5,6]; safety informatics can be employed to promote and improve the safety management of human health, loss prevention and sustainability [7]; a risk-based approach can be used to contribute to the design of an alternative strategy so that the risk resulting from failures can be minimized [8]; and safety investments can be made based on risk-informed decision-making [9]. To develop the quantitative risk assessment (QRA) and dynamic risk assessment of process systems, fuzzy methods, Bayesian networks, bow-tie and other methods can be utilized to make improvements [[10], [11], [12], [13]]. Moreover, the abovementioned methods are valid for addressing uncertainty with respect to the analysis of system safety [14,15]. Developing theoretical models is helpful for the management of safety systems and process safety [[16], [17], [18]]. As a particular research direction of safety science, safety capacity (SC) has been widely applied [19,20]. For instance, SC evaluation models have been utilized to assess the acceptable risk in chemical industrial parks [21]. Furthermore, SC has also been employed in the training of civilian pilots and aviation decision-making processes [22]. However, SC lacks universal generalization, and theoretical research on SC for safety science is ignored. Thus, SC can be studied by summarizing the research methods of system safety.

### 1.2 Literature review

#### 1.2.1 Methods in safety science

Safety science is a cross-discipline that consists of natural science and social science [23]. Safety science studies can be divided into integrated research, cross-sectional studies and disaggregate approaches [24]. Integrated research focuses on systematic study and emphasizes that research should not focus on only one part of the system. Cross-sectional studies can extend research contents based on the study of one point of the system. The disaggregate approach indicates that the system can be divided into subsystems, and research will be conducted for each subsystem. The above three study types can complement each other. Integrality is the purpose, disaggregation is the requirement, and cross-sectional study is the means. As a valid safety research method, safety similarity can be used in cross-sectional studies based on subsystems. Meanwhile, it can also make integrated research for the system based on the safety comparison [25]. Therefore, the concept of capacity can be employed to study SC based on safety comparisons. The current cognition of SC comes from the cognition of capacity, *i.e*., the safety reception threshold.

SC indicates the carrying capacity of system risk, with Safety assessment being at its core. Safety assessment was derived in the insurance industry of the United States (US), and it was developed in the military field. To assess the safety of long-range launches, Bell Labs proposed event tree analysis (ETA) in 1961. With the implementation of corresponding standards by the US Department of Defense (DOD), system safety assessment gained considered development [26]. Moreover, the LEC method was proposed to evaluate operational hazards in potentially hazardous conditions, and it can be combined with probabilistic risk assessment (PRA) to perform semiquantitative safety assessments [27]. In 1964, Dow Chemicals proposed the F&E index method according to its chemical engineering characteristics. In 1974, Imperial Chemical Industries (ICI) proposed an improved method named the ICI MOND. It supplemented the concepts of the toxicity index, safety countermeasures and compensation factor. In 1991, Royal Dutch Shell developed the management system of healthy-safety-environment (HSE), and it has been widely used in the system assessment of the petrochemical industry all over the world [28]. Generally, the framework of safety assessment includes three aspects: (a) Confirmation of the probability. (b) Confirmation of the number of people affected and property damage level. (c) Estimation of the severity of accident consequences.

#### 1.2.2 The development direction of SC

Currently, SC research focuses on the establishment of novel risk assessment methods. For example, Khan and Abbasi developed the accident hazard index (AHI) to comprehensively assess accident risks during the chemical engineering process [29]. The proposed index can enable one to choose between possible sites for setting up a new industry. Leong and Shariff established the inherent safety index module (ISIM) to evaluate the inherent safety level during the preliminary design stage [30]. The proposed module is conducive to the establishment of safer process plants. Fabiano combined risk assessment and decision-making for hazardous material transportation [31]. They proposed a novel assessment module with respect to accident probability and fatality. Chen et al. defined the SC of a chemical industrial park (SCCIP) based on the acceptable regional risk [17]. A corresponding method was adopted for evaluating transport risk and to confirm the SC of chemical industrial parks by using quantitative risk assessment. The results showed that the presented evaluation model can provide effective guidance for the risk control of parks. Wang et al. indicated that the calculation of SC was important for the safety planning of chemical industrial parks [18]. Then, they calculated SC based on the basic information of the park, *i.e.*, individual risk, social risk and potential risk. Meanwhile, the analysis process of the appropriate SC was illustrated using a certain chemical industrial park as a case study. In Luo and Tong's study [32], SC was utilized to assess accidents that occurred in tourist attractions. They indicated that the characteristic of SC is multidimensional, and the maximum SC was confirmed by five dimensions, *i.e.*, substance, environment, psychology, society and economy. However, current studies of SC are limited to the following two aspects: (a) Research on SC is limited to specific fields, including the SC of populations and the SC of hazardous materials. The concepts of SC are confused with the information representation of vulnerable spots. (b) SC is regarded as a safety assessment method. The cognition of connotation with respect to SC has been ignored.

### 1.3 Research goal

This study aims to develop safety capacity, which belongs to the basic theory of safety science. The core concept of safety capacity is refined from the perspective of dimension mining and dimensionality reduction. Based on this fundamental framework, we conduct further investigations into the subprinciple of safety capability, taking into account relevant safety phenomena, thereby strengthening the theoretical foundation of this structure. Additionally, we propose an approach to explore safety capability from the perspective of dimensional attributes. Employing similarity theory, we endeavor to identify new dimensions of risk, continuously striving for higher-dimensional representations of system safety information. Simultaneously, within the comprehensive context of high-dimensional safety information, we utilize dimensionality reduction techniques to transform and simplify these dimensions, allowing for more effective consideration of relevant dimensions and facilitating advancements in system safety.

In this study, the connotation of SC is studied and developed. Section 2 illustrates the theoretical connotation of SC. Then, dimension mining (DM) and dimensionality reduction (DR) are proposed. Corresponding theoretical explorations of them are discussed in Sections 3, 4, respectively.

## 2 Theoretical connotation

**Fig. 1:**
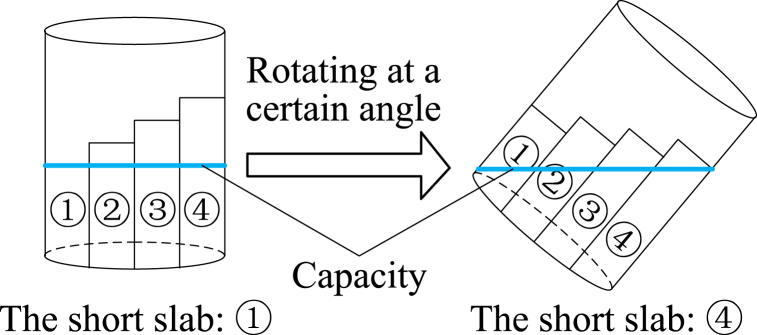
Buckets effect.

To develop a visual illustration of SC, the concept of capacity can be compared with SC to provide an explanation. Generally, capacity indicates the number of objects that an object or space can hold. The critical characteristic of capacity is the amount that can be contained. For example, the bucket effect indicates that the capacity of a container is determined by the short slab (Fig. 1). However, the capacity of the container is dynamic as the container is rotated to different angles. Similarly, a safety system can be regarded as a container, and the safety capacity of the system is determined by the vulnerable spot of the system dimensions.

### 2.1 Dimension attribute of SC

**Fig. 2:**
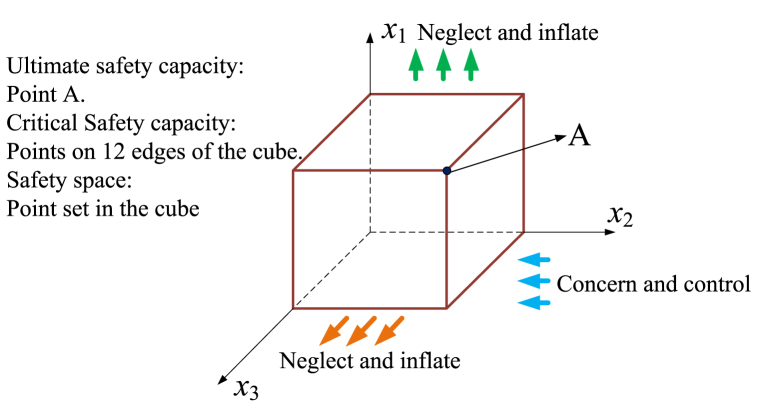
Understanding of SC based on 3-dimensional risk.

From the point of view of safety, capacity consists of safety attributes. For the treatment of capacity based on system safety, the capacity with respect to each dimension in the system can be measured by the system SC. From the point of view of statistics [33], one-dimensional value has a great level of information shortage [34]. Hence, the SC cannot just be presented as a one-dimensional value. The system bearing ability of risk is reflected by SC, and it is determined by all dimensions of risk in the system. Obviously, SC is a multidimensional space vector. Therefore, a more appropriate definition should be made for SC: In a certain system, disturbance caused by people, objects or the environment in different dimensions is allowed. If the system will maintain safety when a series of disturbances reach the maximum value, the allowed maximum value in each dimension can make up the SC, and it is defined as the ultimate SC. In addition, if the maximum value of any dimension and arbitrary value of other dimensions can make up SC, it is defined as critical SC. As an *n*-dimensional space vector, the vector space represents the safety space of the system. Then, SC can be expressed as a vector (Eq. (1)). To make a visual illustration, an idealized system with three dimensions is introduced (Fig. 2).

(1) $${\vec{R}}_{n}=\left(x_{1},x_{2},...,x_{n}\right)$$

where *x* denotes the reception threshold and *n* denotes the number of dimensions.

As shown in Fig. 2, the reception threshold of each dimension reaches the maximum value under the condition of point A. For the condition of points on the 12 edges of the cube, the reception threshold of one dimension can reach the maximum value. In the safety space, all dimensions are within the reception threshold. Safety in one dimension will neglect safety in the other dimensions. Therefore, the key to determining the safety space is the number of dimensions. Some systems have a higher degree; thus, the number of considered risk dimensions is greater. Accordingly, the space dimension of the corresponding SC is higher, that is, the value of *n* is greater. The bounded rationality of safety is reflected in the condition that the dimension number of high-dimensional SC is open.

### 2.2 Research methods of SC

**Fig. 3:**
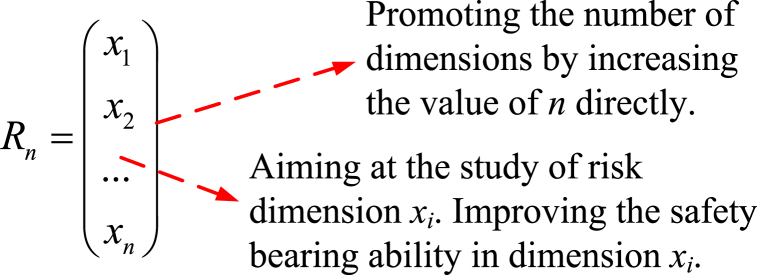
Two research methods for SC.

As previously mentioned, the essence of system SC is system capacity. For the SC, dimension is not limited to any of population, environment, equipment, energy, behavior, etc. A systemic consideration should be made for research on SC. Owing to the uncertainty of the risk dimension and incompatibility of each dimension, systematicness will lead to spatial system capacity [35,36]. As an *n*-dimensional vector, the effectiveness of system safety is determined not only by the value of capacity but also by the possible number of risk dimensions in the system. Hence, two ways are provided to study SC, *i.e.*, DM and improvement of the risk dimension (Fig. 3).

#### 2.2.1 Research on SC from system risk dimensions

**Fig. 4:**
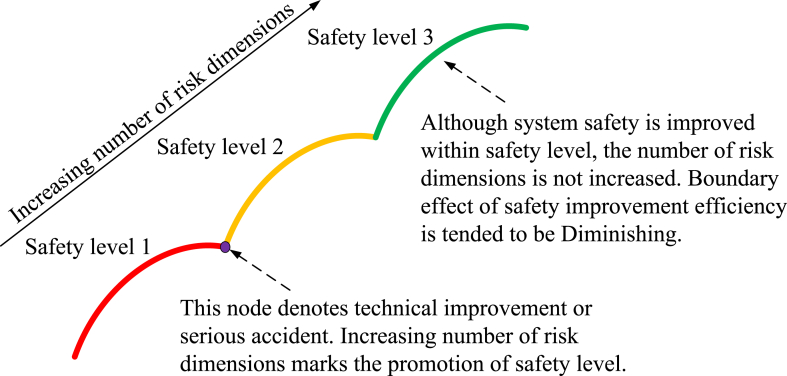
Safety development patterns.

Differences must exist in risk dimensions because SC is an *n*-dimensional vector, and safety awareness for different systems is limited. Hence, system SC needs to correspond to a specific system. Currently, the most universal risk dimensions are man-machine-environment [19,37,38]. It is impossible to obtain all the risk dimensions for SC due to the limitation of awareness for risk. Safety assessment involves relativity and timeliness. System safety levels will be promoted when new risk dimensions are explored. Promoting the number of dimensions with respect to SC will enhance safety. Development patterns are shown in arc-like echelon form (Fig. 4).

As shown in Fig. 4, the DM needs a very large driving force because the awareness of the system is limited by the current state of the art. Compared with the mutation of nodes, most safety studies have taken place on arcs. Therefore, the most important dimension should be found, and its capacity should be promoted. Then, the overall safety of the system can be improved.

**Fig. 5:**
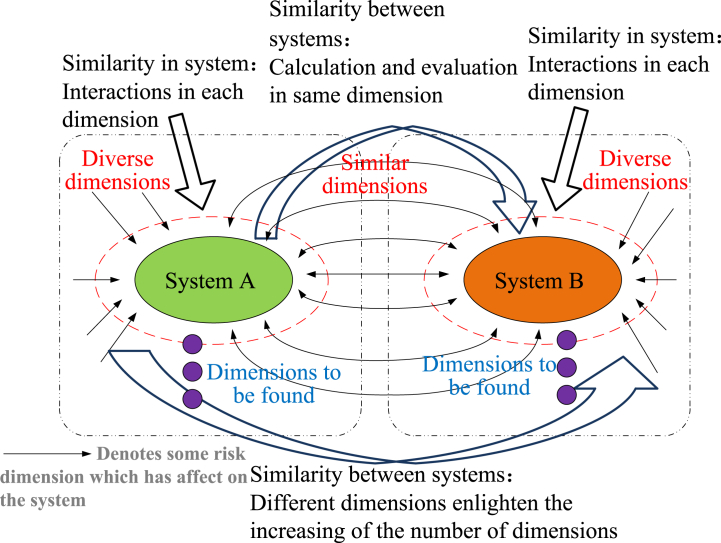
Similarity relations between system dimensions.

Moreover, dimension mining for SC needs to be carried out based on similarity theory [39]. With regard to similarity theory, everything has certain attributes and characteristics. Universal connections are contained in many things; that is, things have common characteristics. Such common characteristics can be called similar characteristics, *i.e*., similarity. Similarity theory has been applied in the study of safety science, including the establishment and application of safety standardization, safety preevaluation according to existing projects, summary of accepted projects, *etc*. [40,41]. Take points, lines and surfaces as examples. Whether it is a point on a line or a line on a surface, the corresponding characteristics have not changed. Clearly, low-dimensional characteristics do exist in the high dimension, and similar characteristics are contained in different vector spaces (Fig. 5).

As shown in Fig. 5, differences in risk dimensions are contained in system A and system B, and they belong to different safety spaces. The number of dimensions can be simplified and normalized based on corresponding standards. Although SC research is convenient, dimensionality reduction must be performed carefully due to the fuzziness of safety representation. Therefore, the employment of similarity theory can avoid different dimensions in different systems. Then, calculation and evaluation in the same dimensions and similar enlightenment in different dimensions can be made.

#### 2.2.2 Research on dimension improvement for safety information

Safety improvement of the system cannot be considered wholly. Hence, the DR is introduced to make selective safety improvements on the relevant dimension (or vulnerable spot). Safety information of high dimensions can be converted into various regnant dimensions, and effective research of SC will be performed. However, dimension attributes cannot be ignored for the effective improvement of system safety. As shown in Fig. 5, limited cognition of safety dimensions must lead to limited safety efficiency. One-sided cognition of SC will be avoided when the risk dimensions are studied as a whole. As a result, research on SC can be enlightened by dimension theory. On the one hand, more risk dimensions should be explored to recognize the system, and comprehensive information on SC needs to be obtained [42]. On the other hand, research should focus on the vulnerable spot of the system to fulfill the requirement of dimensionality reduction.

## 3 Dimension mining and dimensionality reduction

As previously mentioned, SC has dimension attributes. Indeed, the development of safety research is based on the process of DM. With the promotion of risk cognition, new dimensions with respect to safety research are explored. DM for safety theory will contribute to the study of essence for system safety. However, the expanding dimensionality poses formidable challenges for data processing. To address these challenges in safety research, dimensionality reduction techniques are employed as a means of simplifying the dimensions. The concept of safety dimensionality reduction has long been ingrained within the realm of safety research, implicitly woven into contemporary methodologies for studying system safety.

This section will focus on presenting various methods and theoretical concepts related to dimensions mining and dimensionality reduction in safety capacity. The goal is to enhance the understanding of the principles and ideas behind dimension mining and dimensionality reduction.

### 3.1 Principle of analogism in safety research

**Fig. 6:**
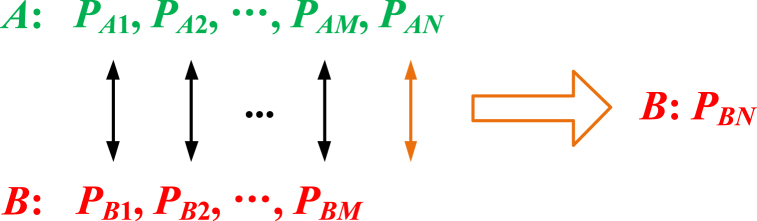
Process of analogism.

Analogism identifies similar attributes of two objects, and the attribute of one object can be confirmed based on the similar attribute of the other object. Assume two objects *A* and *B*. The attributes of *A* and *B* are *P*_*A*1_, *P*_*A*2_, …, *P*_*AM*_, *P*_*AN*_, and *P*_*B*1_, *P*_*B*2_, …, *P*_*BM*_, respectively. Then, the process of analogism is shown in Fig. 6.

To promote the reliability of safety research, more attributes that are lacking in both objects should be listed, and more common attributes need to be explored. The precondition of analogism is that common attributes exist in the analogy objects. Other systems can be confirmed by the known system, which has a higher research degree. The essence of analogism is similar research for objects. Actually, the essence of analogism in safety research is similar research based on similarity theory.

### 3.2 Safety similarity theory

#### 3.2.1 Concept of similar element

Similar characteristics of systems are determined by the essence of common attributes. The presented phenomenon is called the similar phenomenon. In the system, a similar phenomenon corresponds to similar characteristics, and it is named a similar element (*u*) for short. The reason for a similar phenomenon is that similar characteristics have different values, *i.e*., the value of *u* is different. Assuming *n* similar characteristics are contained in the system, *n* similar phenomena are presented. Then, the number of similar elements is *n*. For each similar phenomenon in the system, their values have their ratios. The ratio is called the similar multiple (*c*). Assuming a similar phenomenon has *m* values, similar multiples are *c*_1_, *c*_2_, …, *c*_*m*_ (0＜*c*_*i*_ ≤ 1). Then, the value of similar element *u* is calculated by Eq. (2). If the value of *u* is 0, the two phenomena are neither the same nor similar. The phenomena are similar when the value of *u* belongs to (0, 1), and a greater value of *u* indicates a higher similarity. If the value of *u* is 1, the two phenomena are totally the same.

(2) $$u=\frac{1}{m}\left(c_{1}+c_{2}+...+c_{m}\right)=\frac{1}{m}\sum_{i=1}^{m}c_{i}$$

where *m* is a positive integer.

Assuming the system has *n* similar elements, each phenomenon has *m* values. The *n* similar elements can be confirmed by Eq. (3).

(3) $$u=\left[\begin{array}{l} u_{1}\\ u_{2}\\ ...\\ u_{n} \end{array}\right]=\left[\begin{array}{l} \frac{1}{m}\left(c_{11}+c_{12}+...+c_{1m}\right)\\ \frac{1}{m}\left(c_{21}+c_{22}+...+c_{2m}\right)\\ ...\\ \frac{1}{m}\left(c_{n1}+c_{n2}+...+c_{nm}\right) \end{array}\right]=\left[\begin{array}{l} \frac{1}{m}\sum_{i=1}^{m}c_{1i}\\ \frac{1}{m}\sum_{i=1}^{m}c_{2i}\\ ...\\ \frac{1}{m}\sum_{i=1}^{m}c_{ni} \end{array}\right]$$

Values of similar characteristics will change with time, and the system similarity will also change. At an arbitrary time *t*, the corresponding value of a similar element is *u*(*t*). Assuming the variable time is Δ*t*, then the variation of a similar element is computed by Eq. (4). If the value of Δ*u* is more than 0, the similarity of a similar phenomenon is increasing. In contrast, the similarity of similar phenomena will decrease when the value of Δ*t* is less than 0.

(4) $$\Delta u=u\left(t+\Delta t\right)-u\left(t\right)=\left[\begin{array}{l} u_{1}\left(t+\Delta t\right)-u_{1}\left(t\right)\\ u_{2}\left(t+\Delta t\right)-u_{2}\left(t\right)\\ ...\\ u_{n}\left(t+\Delta t\right)-u_{n}\left(t\right) \end{array}\right]$$

#### 3.2.2 Calculation of system similarity

Assume that the elements of systems *A* and *B* are *a*_1_, *a*_2_, …, *a*_*k*_ and *b*_1_, *b*_2_, …, *b*_*l*_, respectively. Elements in *A* and *B* are expressed as *A* = {*a*_1_, *a*_2_, …, *a*_*k*_} and *B* = {*b*_1_, *b*_2_, …, *b*_*l*_}, respectively. If the element *a*_*i*_ in *A* and the element *b*_*i*_ in *B* have the same characteristics, they can be used to establish a new subset *u* (Eq. (5)). Moreover, *U* denotes *n* subsets when *n* same characteristics existed in the system (Eq. (6)).

(5) $$u=\left\{a_{i},b_{i}\right\}$$

(6) $$U=\left\{u_{1},u_{2},...,u_{n}\right\}$$

Generally, elements in the system have many characteristics. Assuming a similar element has *r* common characteristics, they are expressed as *S*_1_, *S*_2_, …, *S*_*r*_. *U*_*j*_(*a*_*i*_) is set as the characteristic value of element *a*_*i*_ for the characteristic *S*_*j*_. Meanwhile, *U*_*j*_(*b*_*i*_) is set as the characteristic value of element *b*_*i*_ for the characteristic *S*_*j*_. Then, the ratio of the characteristic value *S*_*j*_ with respect to *a*_*i*_ and *b*_*i*_ is calculated by Eq. (7).

(7) $$R_{ij}=\frac{\min\left\{U_{j}\left(a_{i}\right),U_{j}\left(b_{i}\right)\right\}}{\max\left\{U_{j}\left(a_{i}\right),U_{j}\left(b_{i}\right)\right\}}$$

where *R*_*ij*_ denotes the ratio of the characteristic value and *i* and *j* are positive integers.

Because the characteristics have different effects on the elements, the corresponding characteristic weight is introduced. The characteristic weights are set as *d*_1_, *d*_2_, …, *d*_*r*_. Then, the value of similar element *u*_*i*_ within systems *A* and *B* is expressed as *q*(*u*_*i*_), which is confirmed by Eq. (8). If the value of *q*(*u*_*i*_) is equal to 0, the two elements are neither the same nor similar, *i.e.*, there are no common characteristics for them. Two elements are similar when the value of *q*(*u*_*i*_) belongs to (0, 1), with a greater value of *q*(*u*_*i*_) indicating a higher similarity. If the value of *q*(*u*_*i*_) is 1, the two elements are totally the same.

(8) $$q\left(u_{i}\right)=d_{1}R_{i1}+d_{2}R_{i2}+...+d_{m}R_{im}=\sum_{j=1}^{r}d_{j}R_{ij}$$

where *R*_*ij*_ and *d*_*j*_ are within the scope of (0, 1] and [0, 1], and $\sum_{j=1}^{r}d_{j}=1$.

Characteristic weight *d*_*j*_ can be confirmed based on fuzzy methods [43,44]. *K* indices are introduced to judge the characteristic weights (Table 1). The average value of each index weight can be regarded as the characteristic weight (Eq. (9)). Subsequently, the system similarity can be calculated by Eq. (10). If *q*(*u*_*i*_)≡1 and *Q*(*A*, *B*) = 1, the systems will be the same when the values of *k*, *l* and *n* are the same. When the conditions *q*(*u*_*i*_) ≢ 1, *Q*(*A*, *B*) ≢ 1, *k*≠*l*≠*n*, and *Q*(*A*, *B*)∈(0, 1) are satisfied, the systems are similar. A greater value of *Q*(*A*, *B*) indicates a higher similarity. If the value of *n* is equal to 0, the two systems are neither the same nor similar.

(9) $$d_{j}=\frac{1}{K}\sum_{i=1}^{K}d_{ij}$$

(10) $$Q\left(A,B\right)=\frac{n}{k+l-n}\sum_{i=1}^{n}\beta_{i}q\left(u_{i}\right)$$

where *β*_*i*_ denotes the weight of a similar element for the system similarity, and it is also confirmed by expert judgment-based fuzzy methods [45].

**Table 1:** Introduction and classification of accident causation theories.

Index	*S*_1_	*S*_2_	…	*S*_j_	…	*S*_r_
1	*d*_11_	*d*_12_	…	*d*_1*j*_	…	*d*_1*r*_
2	*d*_21_	*d*_22_	…	*d*_2*j*_	…	*d*_2*r*_
…	*…*	…	*…*	…	*…*	*…*
*K*	*d*_*K*1_	*d*_*K*2_	…	*d*_*Kj*_	…	*d*_*Kr*_

### 3.3 Safety similarity theory-based dimension mining

#### 3.3.1 Similar risk dimensions between systems

**Fig. 7:**
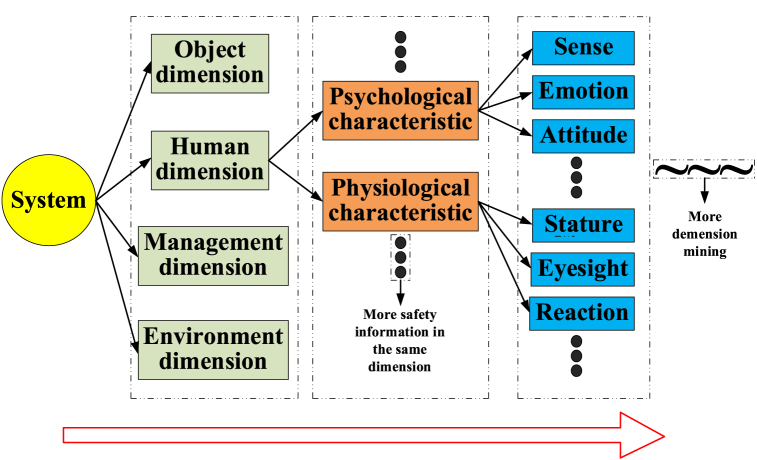
Hierarchy of risk dimension for SC.

Assume that the number of risk dimensions for system *A* is *a* and the number of risk dimensions for system *B* is *b*. Then, their safety capacities are *R*_*a*_=(*x*_1_, *x*_2_, …, *x*_*p*_) and $R_{b}=\left(x_{1}^{0},x_{2}^{0},...,x_{q}^{0}\right)$, respectively. The number of similar risk dimensions is *n*, and the similar element of the risk dimension is expressed as $u=\left\{x_{i},x_{i}^{0}\right\}$. Clearly, the risk in each dimension has its characteristics, and safety assessment results can be the characteristic values. On the one hand, each risk dimension has many safety characteristics. On the other hand, each risk dimension can be characteristic of a higher risk dimension. As the risk dimensions increase, the characteristic on the previous dimension will be the dimension for the next hierarchy (Fig. 7).

Let one risk dimension have *m* safety characteristics. The corresponding safety characteristic weight *d*_*j*_ is confirmed based on the effects of the safety characteristics, and the value of the safety similar element can be calculated by Eq. (11).

(11) $$\left\{ \begin{array}{l} q\left(u_{i}\right)=d_{1}R_{i1}+d_{2}R_{i2}+...+d_{m}R_{im}=\sum_{j=1}^{r}d_{j}R_{ij}\\ 0＜q\left(u_{i}\right)\leq 1 \end{array}\right.$$

Because the same risk dimension is the precondition of a similar safety element, the value of a similar safety element cannot be 0. Similarly, the system will have a higher safety similar degree on the same dimension when the value of *q*(*u*_*i*_) is closer to 1. If the value of *q*(*u*_*i*_) is equal to 1, it will meet the same safety degree. Obviously, the evolution of the system is reflected in the improvement of values of similar characteristics. Safety will be promoted by the replacement of characteristic values on the same dimension and the supplementation of characteristic values on different dimensions.

#### 3.3.2 Similar risk dimensions in the system

**Fig. 8:**
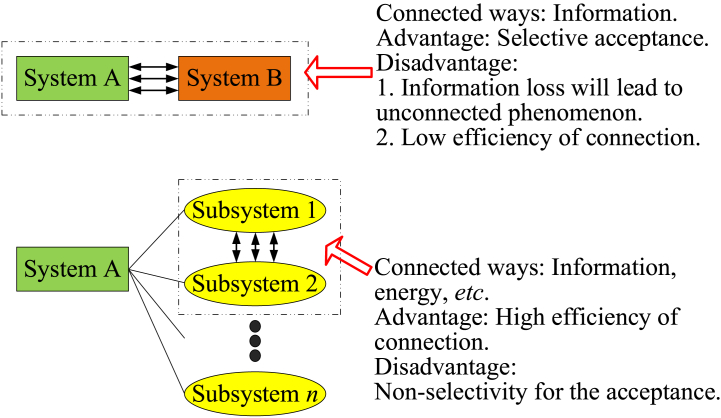
Differences in similar risk dimensions in the system and between systems.

Each risk dimension can be regarded as a subsystem. Hence, research on different risk dimensions in the system can be considered the study of multiple subsystems. Subsystems within the system have complex connections. The connection between different subsystems is not only connected through information but also involves energy connections. Therefore, the worsening of some subsystems will be transmitted to other subsystems because the connection is efficient. The difference in similar risk dimensions in the system and between systems is shown in Fig. 8.

### 3.4 Normalization-based safety assessment

The essence of system safety is related to the DR [46]. Research on system safety includes three aspects, *i.e.*, integrated research, cross-sectional study and disaggregate approach. Therefore, three representative system safety methods are employed to illustrate the implementation of DR.

As an integrated research method, fuzzy evaluation can be used to evaluate the safety of a system. The assessment process is carried out by the following steps.

Step 1 Confirm the set of factors *F* = {*f*_1_, *f*_2_, …, *f*_*m*_} for the assessed system. Meanwhile, safety factors, *i.e.*, risk dimensions, are expressed as *f*_*i*_(*i* = 1, 2, …, *m*). Factor u_i_ must have fuzziness due to the subjectivity in the actual risk dimension.

Step 2 Confirm the set of judgment results *G* = {*g*_1_, *g*_2_, …, *g*_*n*_}. The obtained judgment results are expressed as *g*_*i*_(*i* = 1, 2, …, *n*).

Step 3 Establish the membership matrix, *i.e.*, the fuzzy evaluation matrix. The assessed factor *f*_*i*_ is evaluated, and the corresponding fuzzy vector *Z*_*i*_ for *g*_*i*_ is obtained (Eq. (12)). Then, the fuzzy evaluation matrix can be confirmed (Eq. (13)), and all evaluation information is contained in matrix *Z.*

(12) $$Z_{i}=\left\{Z_{i1},Z_{i2},...,Z_{in}\right\},i=1,2,...,m$$

(13) $$Z=\left[\begin{array}{l} z_{11}\;z_{12}\;...\;z_{1n}\\ z_{21}\;z_{22}\;...\;z_{2n}\\ ...\\ z_{m1}\;z_{m2}\;...\;z_{mn} \end{array}\right]$$

Step 4 Generally, the effects of each safety factor are different. Hence, the weights of each safety factor should be confirmed, and the weight vector *W*=(*w*_1_, *w*_2_, …, *w*_*m*_) is obtained. The weight algorithm can be found in the previous contents in Section 3.2.2.

Step 5 Normalize the judgment results and obtain the final assessment result (Eq. (14)).

(14) $$E=W\times Z=\left(e_{1},e_{2},...,e_{n}\right)$$

The abovementioned result is a simple example of fuzzy evaluation. Evaluation information can be combined with the weight to obtain the evaluation result. Actually, the normalization process of assessment results is the DR of information. Owing to the lack of information caused by DR, assessment results will have confidence coefficients.

### 3.5 Fault tree analysis-based DR

**Fig. 9:**
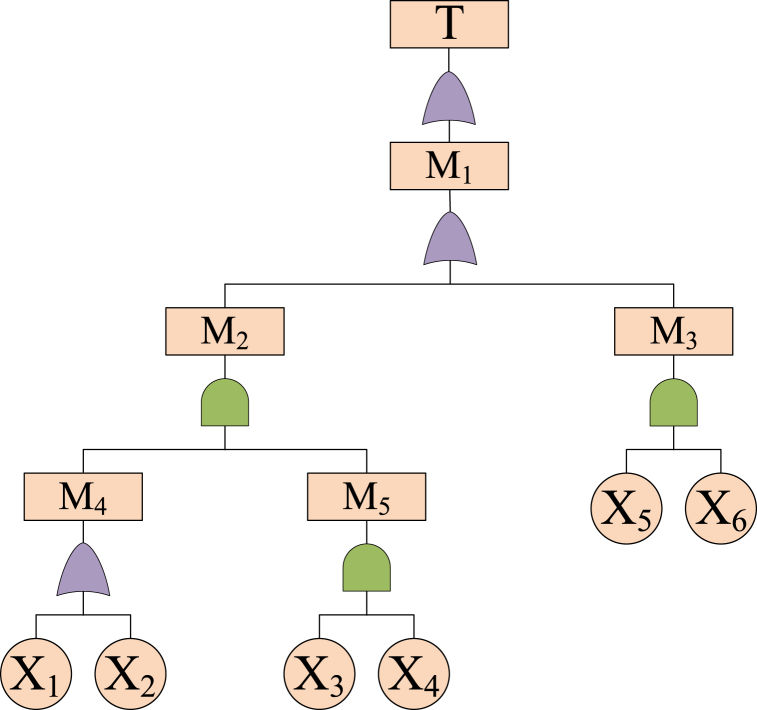
An example of fault tree analysis.

Fault tree analysis is a typical cross-sectional study method. The analysis can be started from the top event, and causes of the top event will be analyzed by the deduction of the fault tree. Moreover, an example of fault tree analysis (Fig. 9) is employed to illustrate the DR process.

Clearly, minimum cut-sets of the top event can be obtained, *i.e.*, *α*_1_ = {X_1_, X_2_, X_5_}, *α*_2_ = {X_1_, X_2_, X_6_}, *α*_3_ = {X_3_, X_5_}, *α*_4_ = {X_3_, X_6_}, *α*_*5*_ = {X_4_, X_5_} and *α*_6_ = {X_4_, X_6_}. The minimum cut-set indicates the minimum quantity of events that will lead to the occurrence of a top event [47]. Then, the structural importance can be calculated by Eq. (15).

(15) $$I_{\varphi \left(i\right)}=1-\prod_{X_{i}\in \alpha_{j}}\left(1-\frac{1}{2^{\theta_{j}-1}}\right)$$

where *I*_*Φ*(*i*)_ denotes the structural importance and *θ*_*j*_ denotes the number of events in the cut-set *α*_*j*_.

The calculated values of structural importance can be ranked as *I*_*Φ(1)*=_ *I*_*Φ*(2)_ < _*IΦ*(3)_ = *I*_*Φ*(4)_ < _*IΦ*(5)_ = *I*_*Φ*(6)_. Then, the ranking of safety improvement is confirmed as X_5_ = X_6_＞X_3_ = X_4_＞X_1_ = X_2_. To achieve the optimal safety efficiency, safety improvement should focus on basic events X_5_ and X_6_. Alternatively, basic events X_5_ and X_6_ can be regarded as the critical nodes of the system. The analysis of critical nodes can reduce the complexity of the assessment process. Obviously, this is a reflection of DR.

### 3.6 DR based on the classification of subsets

**Fig. 10:**
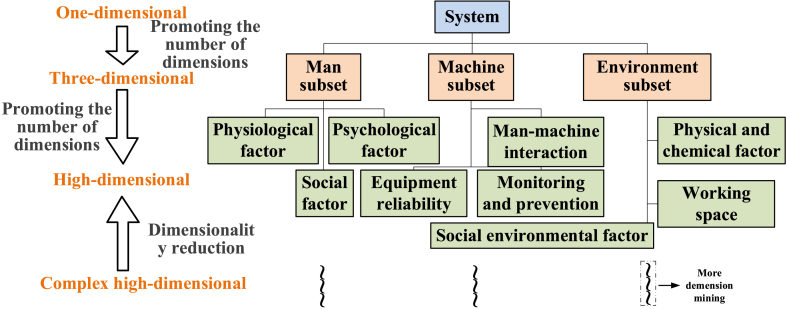
Disaggregation of the man-machine-environment system.

Disaggregation of a complex system can divide it into subsets. The precondition for research on complex systems is the cognition of subsets. Then, the system can be disaggregated, which is similar to the disaggregation of risk dimensions as previously mentioned. On the one hand, the disaggregation of the system can create more dimensions so that more detailed safety information will be contained. On the other hand, the system cannot be disaggregated indefinitely due to the increasing dimensions, which will bring difficulties to data processing. Therefore, the implementation of disaggregation should satisfy the requirement of DR. For example, the man-machine-environment system is a typical cognition of system disaggregation. That is, the system can be divided into three subsets, *i.e.*, the man subset, machine subset and environment subset (Fig. 10).

As shown in Fig. 10, the connection will hardly be described when the system is disaggregated in detail. Hence, the disaggregation of the system needs to address the DR so that the dimensions can be classified.

## 4 Discussion

### 4.1 Threats to validity of dimension mining for safety capacity

**Fig. 11:**
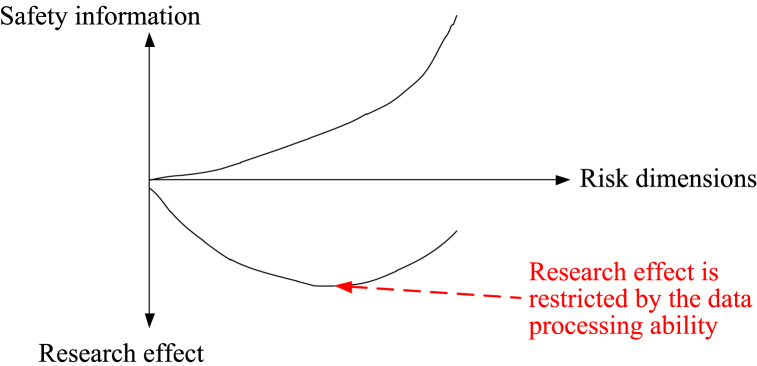
Relationship of safety research effect between risk dimensions and safety information.

Obviously, the implementation of DM will increase safety information. Because SC research is based on the recognition of system risk, the difference in the recognition of risk can lead to different dimensions of SC. The higher recognition ability of risk indicates greater dimensions of SC. With the development of recognition ability, a mass of risk dimensions will be explored. Then, the description of safety information for the system will tend to be impeccable. However, such an ‘explosive increase’ in dimensions leads to difficulty in data processing. If the current data processing ability cannot satisfy the increasing dimensions, the safety research effect will be weakened (Fig. 11).

As shown in Fig. 11, the safety research effect is restricted by the data processing ability while risk dimensions are increasing. Hence, DR is necessary for the improvement of data processing.

On the other hand, the inherent intricacy and diverse challenges encountered in the pursuit of safety research are partially exacerbated by the limitations in data processing capacity entailed by the encompassing high dimensionality of safety information. In practical research endeavors, there arises a need to employ safety dimensionality reduction as a methodological approach to mitigate these challenges, thereby reducing the dimensions of high-dimensional safety information. However, it is paramount to acknowledge that the scope of dimensionality reduction surpasses the mere reduction of risk dimensions. It is necessary with this approach to strictly adhere to the guiding principles of safety dimensionality reduction, which diligently strive to alleviate the complexity associated with data processing while simultaneously endeavoring to uphold the utmost retention of essential safety information. Subsequently, the fourth section will illuminate the conceptual underpinnings of dimensionality reduction.

### 4.2 Application of dimensionality reduction in safety capacity

**Fig. 12:**
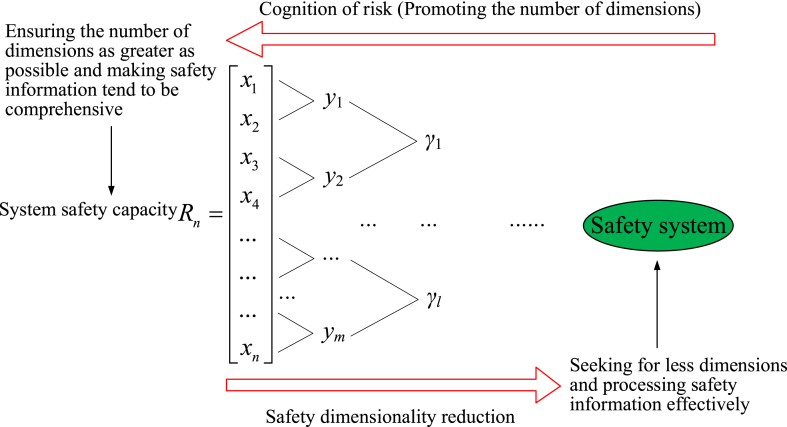
Diagonal pattern for the research of SC.

As previously mentioned, DR is commonly found in safety research. Higher dimensions can contain more comprehensive safety information. Otherwise, DR should be made for high-dimensional safety information due to the limitation of data processing ability. For research on SC, the number of risk dimensions can be confirmed by the cognition of risk. Then, safety information on each dimension is expressed as vectors, *i.e.*, *n*-dimensional SC. More dimensions will contain more comprehensive safety information, and the cognition of system safety will be more rational. However, DR must be implemented due to the limitation of data processing ability. As a key point in the research of SC, the description of DR is shown in Fig. 12.

Actually, DR is just a means for improving safety. It will not contribute much to the promotion of system safety cognition. The greatest significance of safety research is the improvement of safety based on the cognition of system safety, which is the essential goal of safety research. Although improvement of the method with respect to DR can effectively process high-dimensional safety information, the essential cognition of the system still needs continuous dimension mining.

### 4.3 Analysis of boiler water shortage explosion accident in a factory

To demonstrate the practicality of the safety capacity principle, the FTA, a widely used method for dimension mining in safety capacity, was applied to analyze an explosion accident in a factory that was caused by a boiler water shortage. Through this analysis, the role of DR in safety capacity is better illustrated. This method reduced the complexity of the accident, which was originally complex high-dimensional, into a three-dimensional accident, thereby reducing its complexity. By applying this principle, the problem of evaluating accidents that involve excessive safety information can be effectively solved. The analysis of this accident demonstrates the practicality and effectiveness of the DR in safety capacity.

**Fig. 13:**
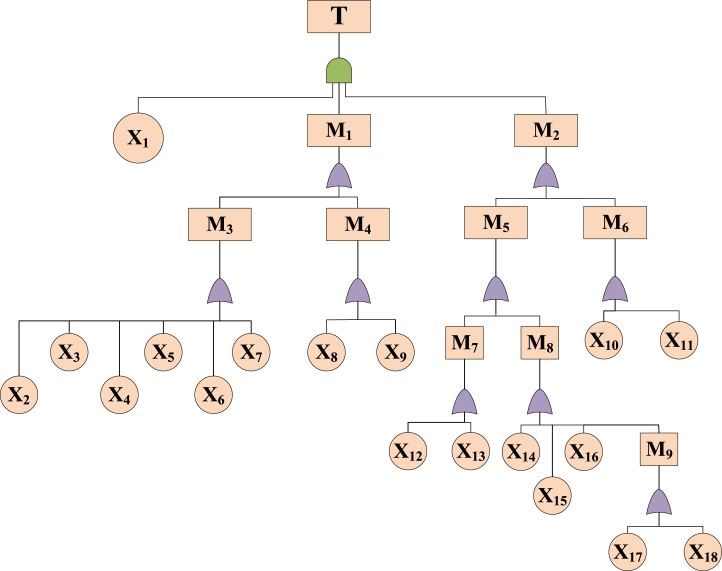
Boiler water shortage accident of the FTA.

By analyzing the causes of the explosion accident that resulted from boiler water shortage, the basic and intermediate events that led to the accident were identified, and the FTA was constructed (Fig. 13, Table 2). Based on the established FTA, we derive the corresponding success tree and compute the structure function as shown below:

$$\bar{T}={\bar{X}}_{1}+{\bar{M}}_{1}+{\bar{M}}_{2}={\bar{X}}_{1}+{\bar{M}}_{3}{\bar{M}}_{4}+{\bar{M}}_{5}{\bar{M}}_{6}={\bar{X}}_{1}+{\bar{X}}_{2}{\bar{X}}_{3}{\bar{X}}_{4}{\bar{X}}_{5}{\bar{X}}_{6}{\bar{X}}_{7}{\bar{X}}_{8}{\bar{X}}_{9}+{\bar{X}}_{10}{\bar{X}}_{11}{\bar{X}}_{12}{\bar{X}}_{13}{\bar{X}}_{14}{\bar{X}}_{15}{\bar{X}}_{16}{\bar{X}}_{17}{\bar{X}}_{18}$$

**Table 2:** Details of FTA Components in Fig. 13.

Symbol	Definition	Description
T	Top event	Boiler water shortage
M1	IE	Water level drawdown
M2	IE	Unaware
M3	IE	Feed water failure
M4	IE	Drain valve failure
M5	IE	Misjudgment
M6	IE	Work fault
M7	IE	Call water error
M8	IE	False water level
M9	IE	Soda cotemp
X1	BE	Alarm failure
X2	BE	Pipeline valve failure
X3	BE	Automatic feed water regulation failure
X4	BE	Cut off the water supply
X5	BE	Pump failure
X6	BE	No steam pump
X7	BE	Cartridge igniter
X8	BE	Valve closed loosely
X9	BE	Open valve
X10	BE	Off duty
X11	BE	Unmonitored
X12	BE	Forget to call for water
X13	BE	Call water is not enough
X14	BE	Water level gauge failure
X15	BE	Not washing regularly
X16	BE	Unreasonable installation of water level gauge
X17	BE	High water hardness
X18	BE	Steam cock closed

**NOTE:** IE = intermediate event; BE = basic event.

According to the structure function, the minimal cut sets of the FTA, namely, *α*_*1*_ = {*X*_*1*_}, *α*_*2*_ = {*X*_*2*_*, X*_*3*_*, X*_*4*_*, X*_*5*_*, X*_*6*_*, X*_*7*_*, X*_*8*_*, X*_*9*_}, and *α*_*3*_ = {*X*_*10*_*, X*_*11*_*, X*_*12*_*, X*_*13*_*, X*_*14*_*, X*_*15*_*, X*_*16*_*, X*_*17*_*, X*_*18*_}, were obtained. Since the accident was relatively simple and did not involve any repeated events, the Minimal Cut Sets could be used to determine the structural importance. Based on this, the structural importance was ordered as follows:

$I_{\theta \left(1\right)}>I_{\theta \left(2\right)}=I_{\theta \left(3\right)}=I_{\theta \left(4\right)}=I_{\theta \left(5\right)}=I_{\theta \left(6\right)}=I_{\theta \left(7\right)}=I_{\theta \left(8\right)}=I_{\theta \left(9\right)}>I_{\theta \left(10\right)}=I_{\theta \left(11\right)}=I_{\theta \left(12\right)}=I_{\theta \left(13\right)}=I_{\theta \left(14\right)}=I_{\theta \left(15\right)}=I_{\theta \left(16\right)}=I_{\theta \left(17\right)}=I_{\theta \left(18\right)}$ Subsequently, the advantages of safety improvements are ranked as follows:

$$X_{1}>X_{2}=X_{3}=X_{4}=X_{5}=X_{6}=X_{7}=X_{8}=X_{9}>X_{10}=X_{11}=X_{12}=X_{13}=X_{14}=X_{15}=X_{16}=X_{17}=X_{18}$$

Furthermore, within the realm of system research, the exclusion of disadvantageous basic events (e.g., *X*_*10*_*, X*_*11*_*, X*_*12*_*, X*_*13*_*, X*_*14*_*, X*_*15*_*, X*_*16*_*, X*_*17*_*, X*_*18*_) based on their significance while focusing on advantageous basic events (e.g., *X*_*1*_) becomes essential for achieving efficient enhancements in system safety. This selective disregard enables targeted attention to vulnerable areas of the system, thereby facilitating more effective research strategies. Nevertheless, it is important to note that such disregard is not absolute, as system improvements inherently result in alterations to the importance of basic events. Hence, the study of weak areas should consider the relative nature of the research objects, necessitating a systematic approach to safety research.

Through the application of the FTA, the identification of weak areas within the system, the elimination of factors with minimal impact on system safety, and the reduction in analytical complexity collectively facilitate efficient safety research, effectively exemplifying the concept of dimensionality reduction.

the approach outlined in this research selectively (and temporarily) excludes disadvantaged dimensions while prioritizing weak areas. By relinquishing low-weighted safety information in the system at the outset, this approach enables targeted allocation of limited resources toward addressing more impactful safety information, making it a fundamental method for enhancing system safety.

The comprehensive examination of a boiler explosion resulting from water shortage in an industrial facility presented herein serves as a tangible exemplification of the pragmatic implementation of safety capacity (SC) in accident prevention, emphasizing the cruciality of employing dimensionality reduction (DR) to streamline intricate accident scenarios. By illustrating this specific incident, readers gain enhanced insight into the practical application of SC, comprehending its potential to fortify safety measures and diminish the probability of accidents across diverse contexts.

## 5 Summary and conclusion

In this research, the theoretical connotation of SC has been defined and developed. Bounded rationality-based research shows that SC has dimension attributes, and therefore, two research directions, *i.e.*, the DM and DR of SC, are proposed. Several theoretical and practical contributions were made: (a) Research on SC was integrated. The dimension attributes of SC were defined. (b) Bounded rationality of safety was proposed. It was found that accident bias existed in the safety research. (c) The safety similarity theory was proposed and defined. The feasibility of DM was demonstrated based on safety similarity theory. (d) Several typical methods with respect to DR were summarized. Safety research can take advantage of DR to process safety information. (e) A diagonal pattern of safety research was proposed. SC should explore the essence of the system by the DM. (f) This study employs the FTA method to examine the occurrence of boiler explosions resulting from water shortage, with the aim of explicating the inherent DR associated with this incident. The primary objective is to furnish readers with a thorough comprehension of the dimensionality reduction concept within the domain of SC. Meanwhile, SC should also improve system safety by DR. This paper comprehensively integrates the research on SC, endowing it with dimensional attributes. The theoretical essence of SC is clearly defined. Based on this definition, two distinct research directions concerning SC are elucidated: the dimension aspect (study of DM) and the dimensionality aspect (study of DR). It's important to note that this paper confines its exploration to the theoretical realm of safety capacity and its related research, without introducing novel techniques or methodologies. In the realm of SC research, potential future studies could involve the integration of DR with big data, facilitated by computational programming algorithms for the processing of intricate safety information.

## Data availability statement

The research subject of this paper does not entail the utilization of extensive datasets. The data involved primarily comprises publicly available literature, texts, or relevant citation materials, all of which have been entirely referenced with explicit source attributions. Furthermore, substantial experimental data, survey data, or any large-scale datasets necessitating public sharing were not employed in this paper.

## Additional information

No additional information is available for this paper.

## CRediT authorship contribution statement

**Fang Yan:** Conceptualization, Formal analysis, Funding acquisition, Investigation, Validation, Visualization, Writing – original draft, Writing – review & editing. **Xuan Li:** Formal analysis, Investigation, Methodology, Validation, Visualization. **Bing Wang:** Formal analysis, Investigation, Methodology, Resources. **Youxian Xie:** Formal analysis, Investigation, Validation, Visualization. **Chao Wu:** Formal analysis, Methodology, Supervision.

## Declaration of competing interest

The authors declare that they have no known competing financial interests or personal relationships that could have appeared to influence the work reported in this paper.
